# Dr. Kinglake's Reply to Mr. Aber

**Published:** 1813-08

**Authors:** Robert Kinglake

**Affiliations:** Taunton


*To the Editors of the Medical and Physical Journal.*

GENTLEMEN,

MY respectful acknowledgment is due to your correspondent Mr. Aber, for the reference he has given me, in the last Number of your Journal, to Mr. Barrett's publication on the analogy subsisting between Hydrophobia and Gastritis. In that publication, as Mr. Aber truly observes, I have been certainly anticipated, both in my theoretical and practical speculations on the subject. It is indeed possible that the remarks alluded to may at the time have been read by me, but I have most assuredly not the smallest recollection of that having been actually the case. At that period, however, the perusal would not have probably made any very strong impression on my mind, as neither direct nor analogical experience had then afforded the subject the degree of interest in my estimation which subsequent occurrences have since given it. Although Mr. Barrett's observations, if ever known to me, had totally escaped my remembrance, yet it is still conceivable that they may have furnished a clue for noticing the resemblance which appeared to me to obtain between Gastritis and Hydrophobia. Of the latter disease I have had no practical knowledge, but the ample details given of it by other medical observers, enabled me to liken it to the appearances presenting in Gastritis, and seemed to warrant me in inferring, from the apparent similitude between the two diseases, that considerable similarity of cause must also necessarily exist to produce such corresponding effects. Mr. Barrett, in his publication, rests the supposed resemblance between Hydrophobia and Gastritis on the morbid appearance in the œsophagus, and in the cardiac portion of the stomach, discovered by dissection, in the the former disease. My views on the subject arose from the symptoms and treatment of the latter affection; Mr. Aber has therefore justly observed, that Mr. Barrett's paper is the more valuable of the two. The deficiency of my speculation seems to have been abundantly supplied by the comprehensive statement of facts in Mr. Barrett's narrative. The practical induction too, as to the most efficient remedy, that of copious bleeding, is circumstantially proposed and enjoined by Mr. Barrett. Had I been aware of Mr. Barrett's paper on the subject referred to, it would have afforded me infinitely more gratification to have supported his judicious opinion by my experience of the advantages of bleeding in Gastritis, and by my persuasion of the correctness of the alleged affinity between the two diseases, than to have offered to the attention of the public observations that were much too crude and incomplete to be entitled to unqualified admission.

The coincidence that has occurred between Mr. Barrett and myself, is perhaps fortunate for the farther investigation of the subject. It may have the useful effect of bringing the question again into discussion, and of obtaining a stock of facts and observations that may be conclusive on the merits of the inquiry. On no other occasion has bleeding ever by my direction been carried to the length that it was in the case of Gastritis formerly recited, and the ultimate success of the treatment in that instance appeared to be closely connected with its *frequent* repetition during a short period. In my judgment, it would appear to be a good general rule in inflammatory affections, to draw blood not as is most commonly done *daily*, or *twice* a-day, but to repeat it every *two* or *three* hours whilst indicated by either morbid tone and hardness of vascular action, by inordinate visceral excitement, or by acute pain. The object in extensively deplenishing the vascular system, is to lessen the stimulus of distention, and to diminish the contractile power of the muscular fibre to a degree that would incapacitate the inflamed parts for sustaining the inflammatory action. This effect cannot be accomplished by insufficient means; the cause must be equal to the desired effect. The course of *anti-inflammant* bleeding, that should be instituted in diseases like Hydrophobia and Gastritis, should be limited only by that degree of vital energy that would be secure against the extinction of life. Within this limit much freedom may be safely taken. When an evacuation of blood has rendered the action of the heart slow and feeble, even to *deliquium*, it will be discreet to desist, though indeed in this prudential forbearance nature commonly anticipates what is necessary by rendering it impossible to carry the discharge farther, until the contractile power of the heart and arteries be somewhat restored. The interval for effecting this renewal of power, is that which should determine the repetition of bleeding in inflammatory affections possessing any kindred violence to hydrophobia and gastritis, and this will in general be found to be about *three* hours rather than *twelve* or *twenty-four*, as usually enjoined and acted on. I am firmly persuaded that a more valid and useful rule could not be proposed in the cure of visceral inflammation, than that of drawing blood every *three* hours, in such quantities at each time as the action of the heart will bear, and continuing the practice as long as the characteristic symptoms of inflammatory disease may remain.

Would not phrenitis, pleuritis, pneumonia, carditis, hepatitis, enteritis, cystitis, and other similar affections, be at least equally benefited by such treatment as hydrophobia and gastritis? Experience, the unerring touchstone of truth, will surely answer in the affirmative.

*Taunton,* I am, &c.

*June* 14, 1813. ROBERT KINGLAKE.

